# P01-011 – Colchicine compliance and amyloidosis

**DOI:** 10.1186/1546-0096-11-S1-A15

**Published:** 2013-11-08

**Authors:** H Ozdogan, S Ugurlu, G Hatemi

**Affiliations:** 1 Department of Internal Medicine, Division of Rheumatology, Cerrahpasa Medical Faculty, University of Istanbul, Istanbul, Turkey

## Objectives

To assess colchicine compliance in patients with familial Mediterranean fever and amyloidosis prior to the development of amyloidosis.

## Methods

Twenty-six patients with FMF amyloidosis were questionned for disease onset, date of diagnosis for FMF and amyloidosis, delay in diagnosis, colchicine dose, response, compliance, disease manifestations, family history, and associated diseases.

## Results

In 14 of the 26 patients, FMF and amyloidosis were diagnosed at the same time with a mean delay in diagnosis of 22 ±9.2 years. In the remaining 12, there was a mean delay of 9.6±8 years from the onset to the diagnosis of FMF and 23±9.6 years from the onset to the diagnosis of amyloidosis. These patients were on colchicine for a mean of 13±7.6 years after the diagnosis of FMF. Eight were non-compliant, however 4 were compliant and recieved 1.5 mg/day of colchicine for a mean of 7.5 years (range 4-12 years) before the development of amyloidosis. One of these 4 compliant patients stopped colchicine 1 year prior to the diagnosis of amyloidosis after 12 years of treatment. Response to Colchicine was reported in 3 patients. History of amyloidosis was present in one and history of FMF in 3 of the 4 compliant patients. None had an associated disease. Two were homozygous and one was heterozygous for M694V.

## Conclusion

This retrospective data may indicate that in a proportion of patients with FMF who had recieved a proper dose of Colchicine can still develop amyloidosis. This observation deserves to be tested in a larger group of patients with FMF amyloidosis.

## Disclosure of interest

None declared

